# Supplementary material for: Cardiac Fibroblasts regulate myocardium and coronary vasculature development via the collagen signaling pathway
Source: bioRxiv. 2025 Mar 20:2024.09.11.612512. Originally published 2024 Sep 12. Preprint. [Version 2] doi: 10.1101/2024.09.11.612512 (PMC11418987; doi:10.1101/2024.09.11.612512)
Supplement: Supplement 1 — Supplementary Table 1: Primer sequences for PLISH probes [file media-1.docx]

| **Name** | **Sequence** |
| --- | --- |
| cy5-mCol1a1-Right-1 | CCACCCCTTCACAGAGATGTTTATACGTCGAGTTGAACGTCGTAACA |
| cy5-mCol1a1-left-1 | TAGCGCTAACAACTTACGTCGTTATGAGCACCTTTGATACCAAACT |
| cy5-mCol1a1-Right-2 | ACACAATTGCACTGAGGAATTTATACGTCGAGTTGAACGTCGTAACA |
| cy5-mCol1a1-left-2 | TAGCGCTAACAACTTACGTCGTTATGAGAACGGTCTCTCCCACCCA |
| cy5-mCol1a1-Right-3 | CATGGAGATGCCAGATGGTTTTATACGTCGAGTTGAACGTCGTAACA |
| cy5-mCol1a1-left-3 | TAGCGCTAACAACTTACGTCGTTATGAGGTTCCTTCAACAGTCCAA |
| cy5-mCol1a1-Right-4 | GACTTATACCCACATAGGTCTTATACGTCGAGTTGAACGTCGTAACA |
| cy5-mCol1a1-left-4 | TAGCGCTAACAACTTACGTCGTTATGTTCAAGCAAGAGGACCAAGC |
| cy5-mCol1a1-Right-5 | GCCCCAAGTTCCGGTGTGACTTATACGTCGAGTTGAACGTCGTAACA |
| cy5-mCol1a1-left-5 | TAGCGCTAACAACTTACGTCGTTATGTCGTGCAGCCGTCCACAAGG |
