# Supplementary material for: Cardiac Fibroblasts regulate myocardium and coronary vasculature development via the collagen signaling pathway
Source: bioRxiv. 2025 Mar 20:2024.09.11.612512. Originally published 2024 Sep 12. Preprint. [Version 2] doi: 10.1101/2024.09.11.612512 (PMC11418987; doi:10.1101/2024.09.11.612512)
Supplement: 10 — Supplementary Fig. S1: (A) Analysis of fibroblast anatomical pattern by staining Col1a1 at different stages. Scale bar=500µm. Supplementary Fig. S2: (A, B) RNA staining of Col1a1, Actn2, and Cd31 expression at E13.5 and P3 heart sections. Scale bar=500µm and 100µm in the whole heart sections and enlarged sections, respectively. Supplementary Fig. S3: ScRNA-seq identified distinct fibroblast populations. (A) UMAP plot of Col1a1 expression in cardiac cells. (Bi-iv) UMAP plots of Col1a1 and representative cluster-specific genes expression in cardiac fibroblasts. (C) UMAP plot of fibroblasts labeled by cell cycle phases. (D) Diagram of the four types of cardiac fibroblasts. (E) The top 10 genes expressed in each group of cardiac fibroblasts. (Fi-iii) Identification of the anatomical location of each fibroblast population through RNA staining. Scale bar=500µm and 250µm in the whole heart images and the images with enlarged areas, respectively. Supplementary Fig. S4: Heterogeneity analysis of cardiac fibroblasts. (A, B) The cell types and main_fb population in CD1 scRNA-seq dataset. (C, D) The cell types and main_fb population in C57BL/6 scRNA-seq dataset. (E, F) The main_fb cells labeled by stage and chamber. Supplementary Fig. S5. The expression pattern of extracellular matrix genes in the main population of cardiac fibroblasts. (A) Clustering analysis of the ECM gene expression enrichments. (B) The group of genes (G1) that were highly expressed at early staged fibroblasts in all four zones. (C) The group of genes (G2) that were highly expressed in LV and RV at late embryonic and neonatal stages. (D) The genes (G3) that were preferentially expressed in LA and RA. (E) The genes (G4) that were highly expressed in LA, LV, and RV at neonatal stage. Supplementary Fig. S6: (A, B) The expression pattern of a group of genes (G5) that displayed expression in all four chambers of FBs (CD1 mice) at late embryonic and neonatal stages. Supplementary Fig. S7: The expression pattern a [file NIHPP2024.09.11.612512v2-supplement-10.pdf]

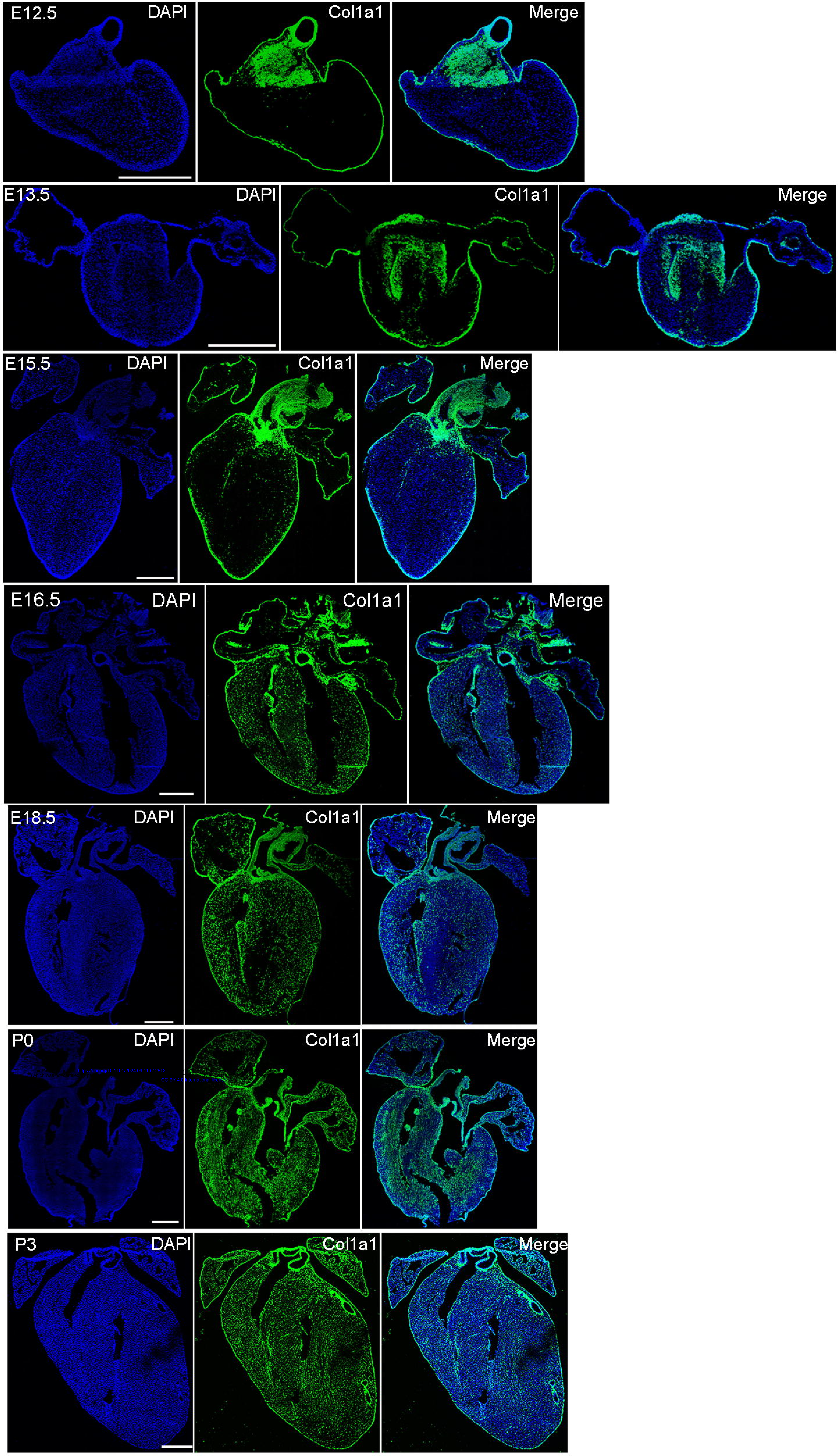
E12.5
DAPI
Col1a1
Merge
E13.5
DAPI
Col1a1
Merge
E15.5
DAPI
Col1a1
Merge
E16.5
DAPI
Col1a1
Merge
E18.5
DAPI
Col1a1
Merge
P0
DAPI
Col1a1
Merge
P3
DAPI
Col1a1
Merge

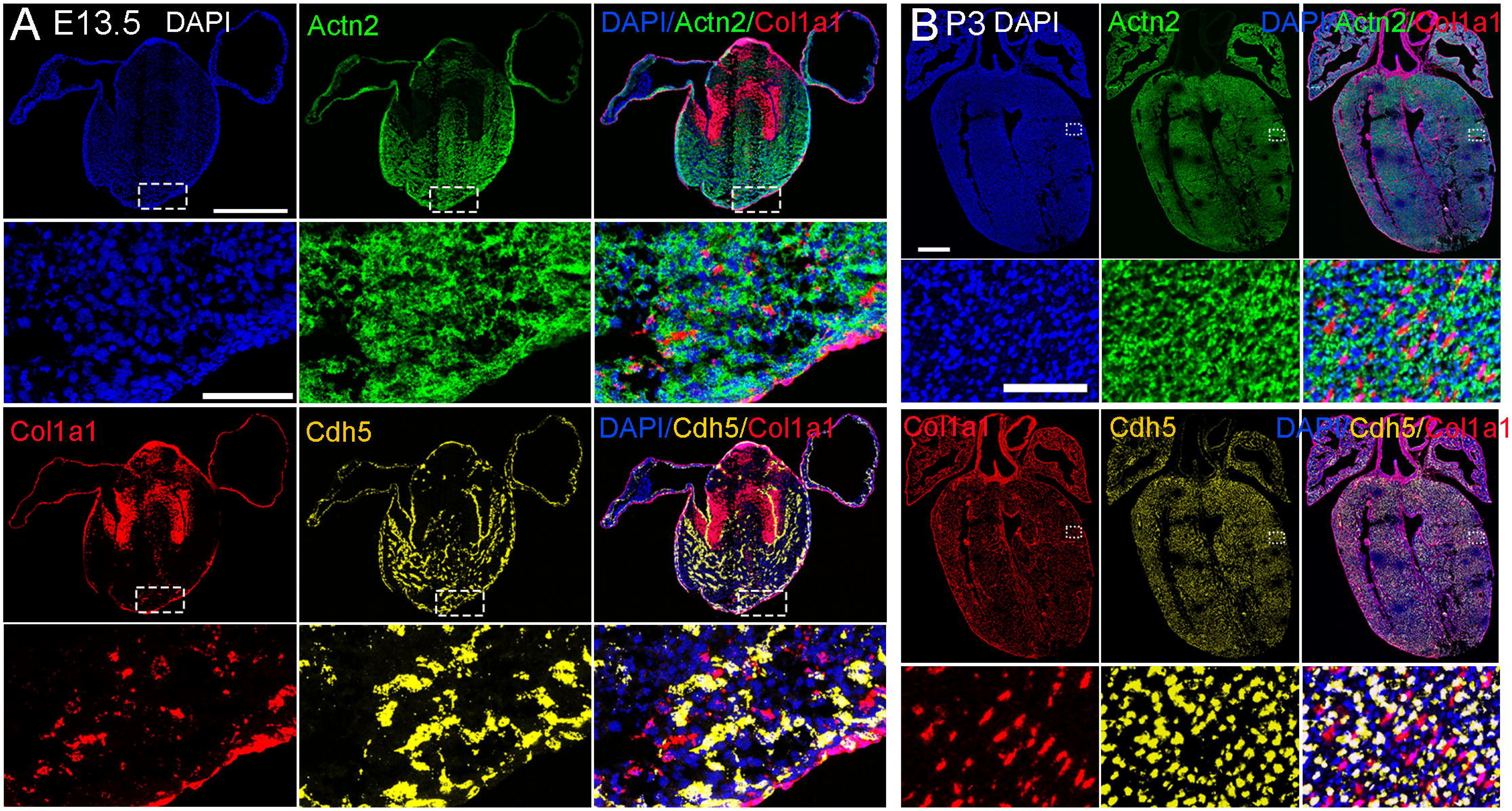

bioRxiv preprint doi: https://doi.org/10.1101/2024.09.11.612512; this version posted March 20, 2025. The copyright holder for this preprint (which was not certified by peer review) is the author/funder, who has granted bioRxiv a license to display the preprint in perpetuity. It is made available under aCC-BY 4.0 International license.

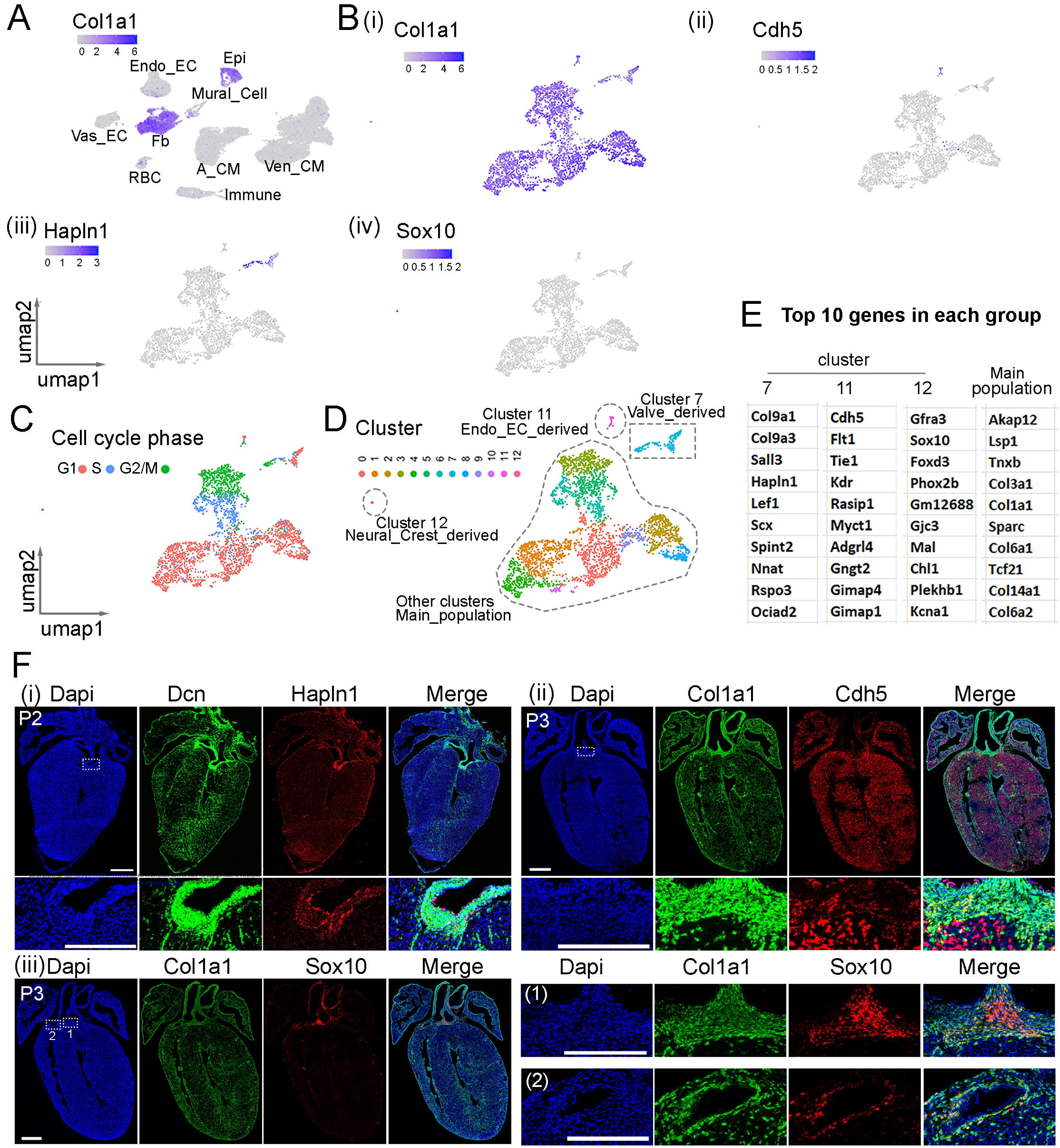
A
Col1a1
0 2 4 6
Endo_EC
Epi
Mural_Cell
Vas_EC
Fb
RBC
A_CM
Ven_CM
Immune
B (i)
Col1a1
0 2 4 6
(ii)
Cdh5
0 0.5 1 1.5 2
(iii) Hapln1
0 1 2 3
(iv) Sox10
0 0.5 1 1.5 2
umap2
umap1
C
Cell cycle phase
G1 S G2/M
umap2
umap1
D
Cluster
0 1 2 3 4 5 6 7 8 9 10 11 12
Cluster 11
Endo_EC_derived
Cluster 7
Valve_derived
Cluster 12
Neural_Crest_derived
Other clusters
Main_population
E
Top 10 genes in each group
cluster
7
11
12
Main population
Col9a1 Cdh5 Gfra3 Akap12
Col9a3 Flt1 Sox10 Lsp1
Sall3 Tie1 Foxd3 Tnxb
Hapln1 Kdr Phox2b Col3a1
Lef1 Rasip1 Gm12688 Col1a1
Scx Myct1 Gjc3 Sparc
Spint2 Adgrl4 Mal Col6a1
Nnat Gngt2 Chl1 Tcf21
Rspo3 Gimap4 Plekhb1 Col14a1
Ociad2 Gimap1 Kcna1 Col6a2
F
(i) Dapi
Dcn
Hapln1
Merge
P2
(ii) Dapi
Col1a1
Cdh5
Merge
P3
(iii)Dapi
Col1a1
Sox10
Merge
P3
2
1
Dapi
Col1a1
Sox10
Merge
(1)
(2)

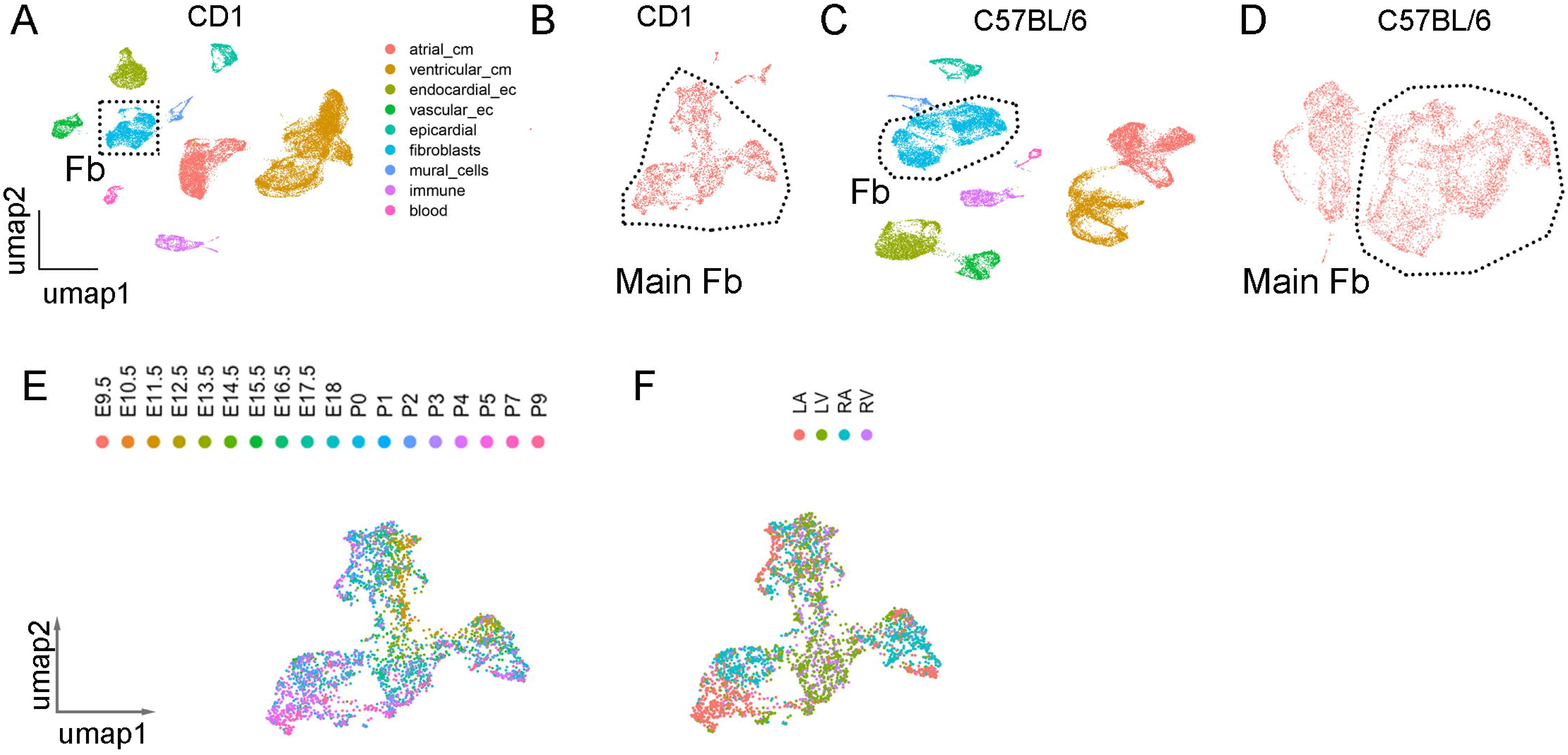

bioRxiv preprint doi: https://doi.org/10.1101/2024.09.11.612512; this version posted March 20, 2025. The copyright holder for this preprint (which was not certified by peer review) is the author/funder, who has granted bioRxiv a license to display the preprint in perpetuity. It is made available under aCC-BY 4.0 International license.

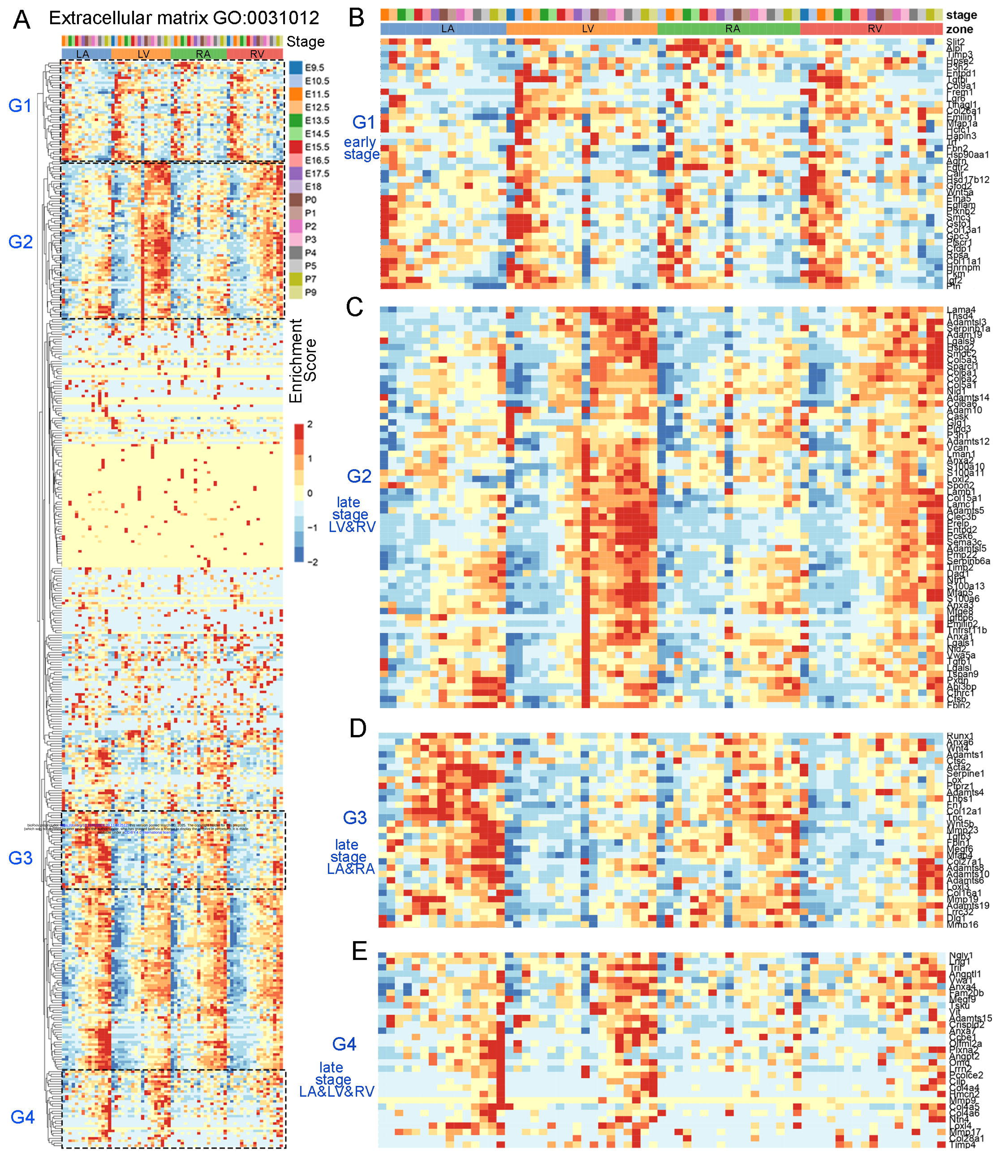

bioRxiv preprint doi: https://doi.org/10.1101/2024.09.11.612512; this version posted March 20, 2025. The copyright holder for this preprint (which was not certified by peer review) is the author/funder, who has granted bioRxiv a license to display the preprint in perpetuity. It is made available under aCC-BY 4.0 International license.

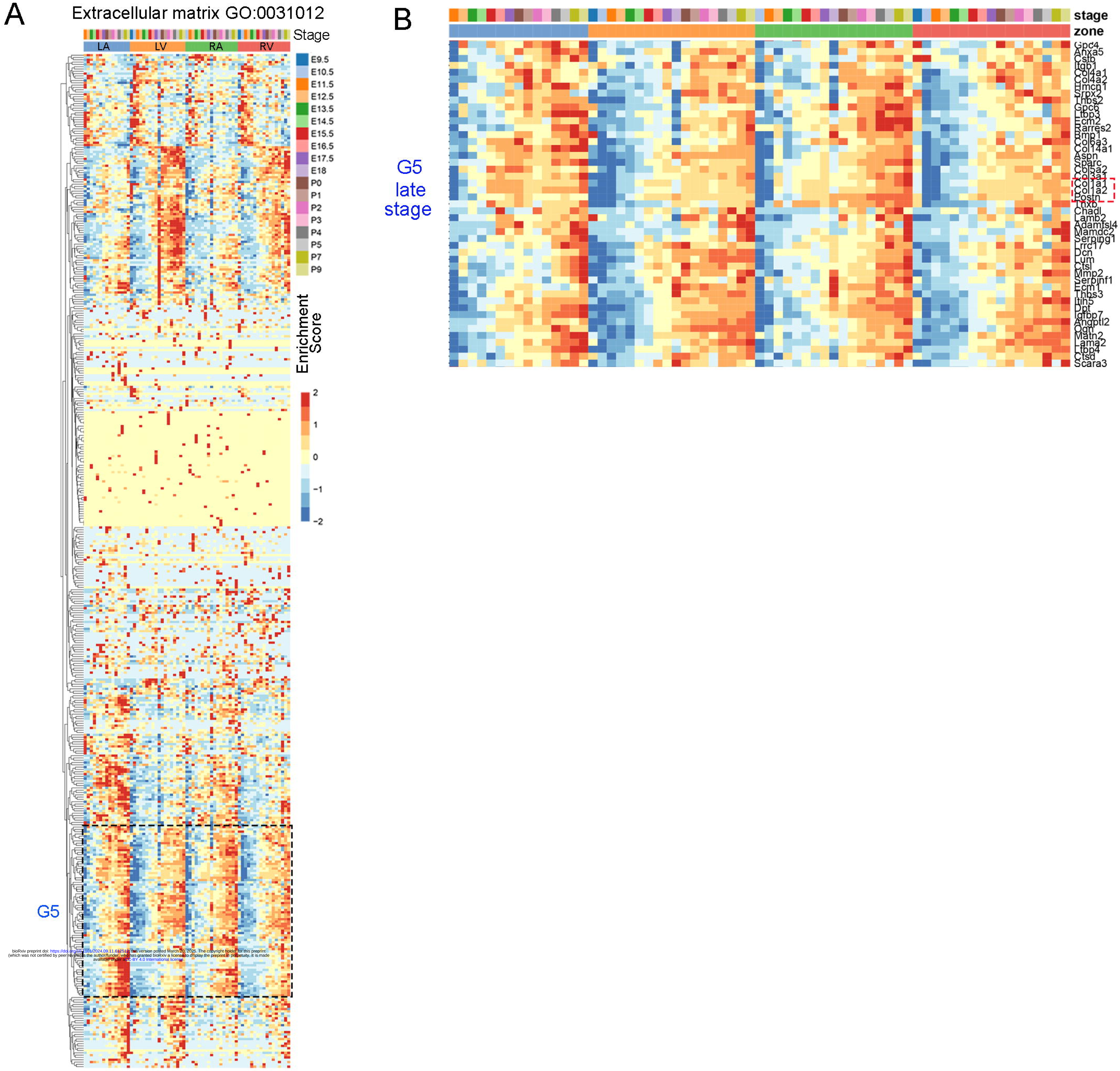

bioRxiv preprint doi: https://doi.org/10.1101/2024.09.11.612512; this version posted March 20, 2025. The copyright holder for this preprint (which was not certified by peer review) is the author/funder, who has granted bioRxiv a license to display the preprint in perpetuity. It is made available under aCC-BY 4.0 International license.

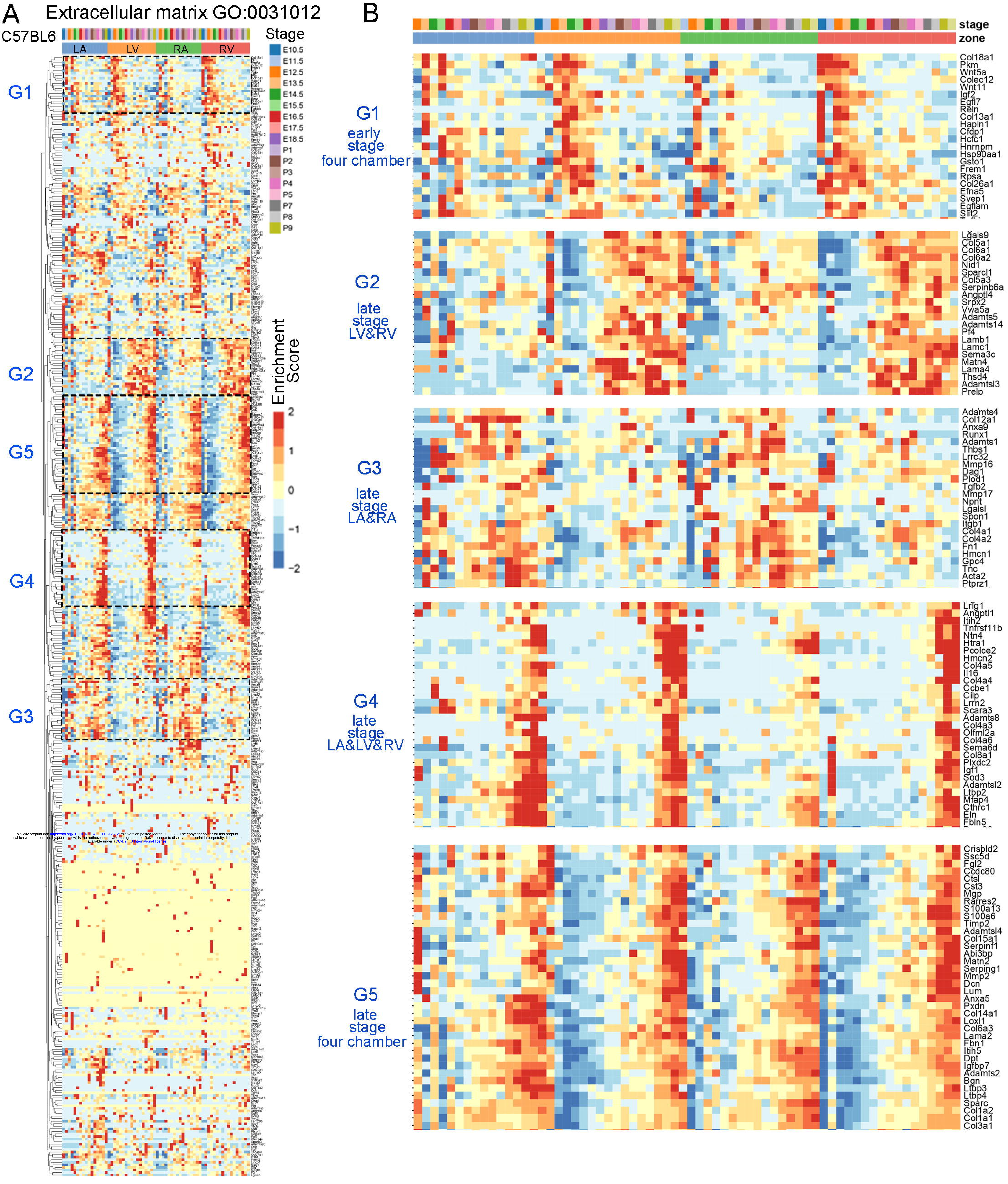

bioRxiv preprint doi: https://doi.org/10.1101/2024.09.11.612512; this version posted March 20, 2025. The copyright holder for this preprint (which was not certified by peer review) is the author/funder, who has granted bioRxiv a license to display the preprint in perpetuity. It is made available under aCC-BY 4.0 International license.

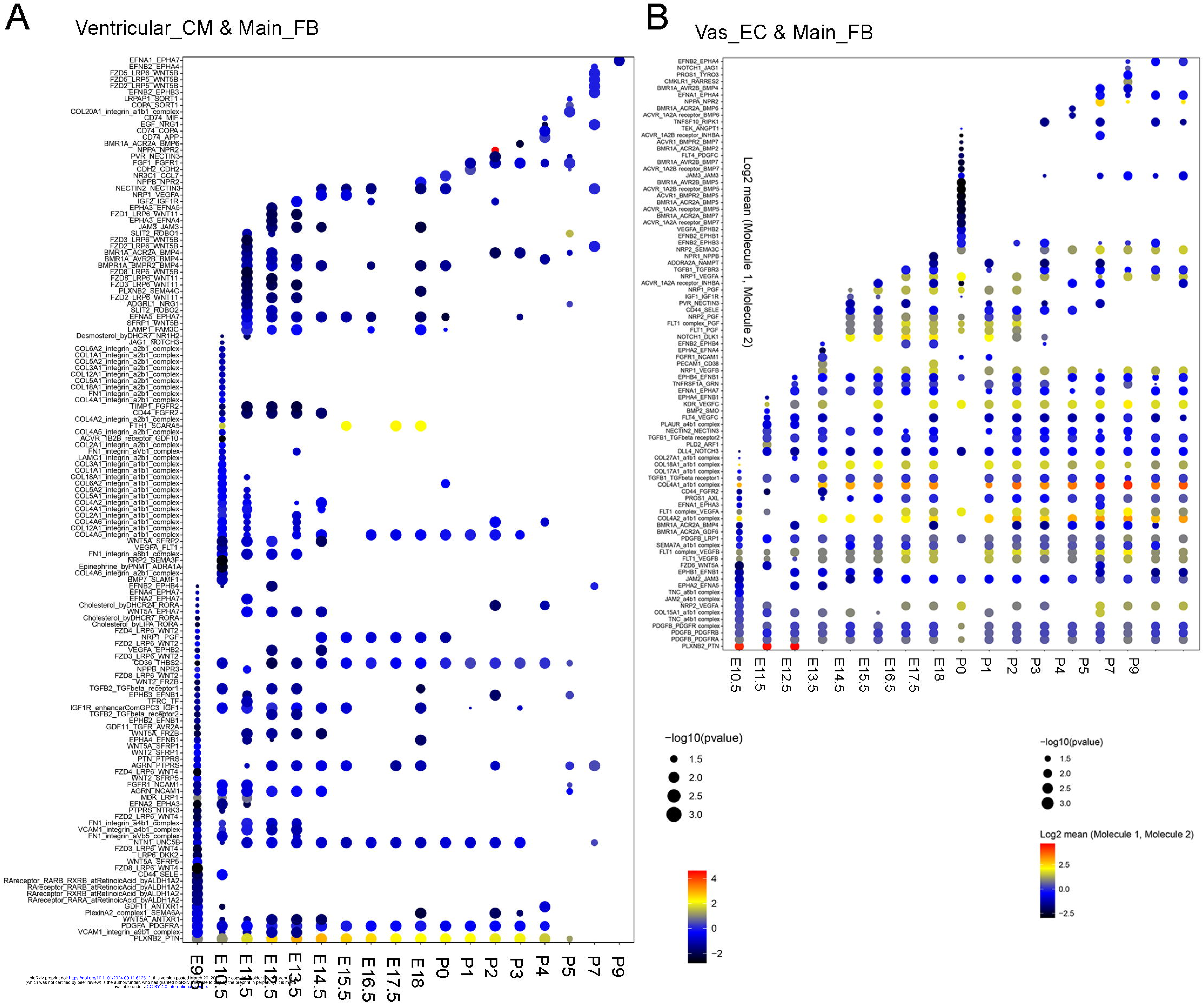
A
Ventricular_CM & Main_FB
E9.5
E10.5
E11.5
E12.5
E13.5
E14.5
E15.5
E16.5
E17.5
E18
P0
P1
P2
P3
P4
P5
P7
P9
B
Vas_EC & Main_FB
Log2 mean (Molecule 1, Molecule 2)
E10.5
E11.5
E12.5
E13.5
E14.5
E15.5
E16.5
E17.5
E18
P0
P1
P2
P3
P4
P5
P7
P9
−log10(pvalue)
1.5
2.0
2.5
3.0
4
2
0
−2
−log10(pvalue)
1.5
2.0
2.5
3.0
Log2 mean (Molecule 1, Molecule 2)
2.5
0.0
−2.5

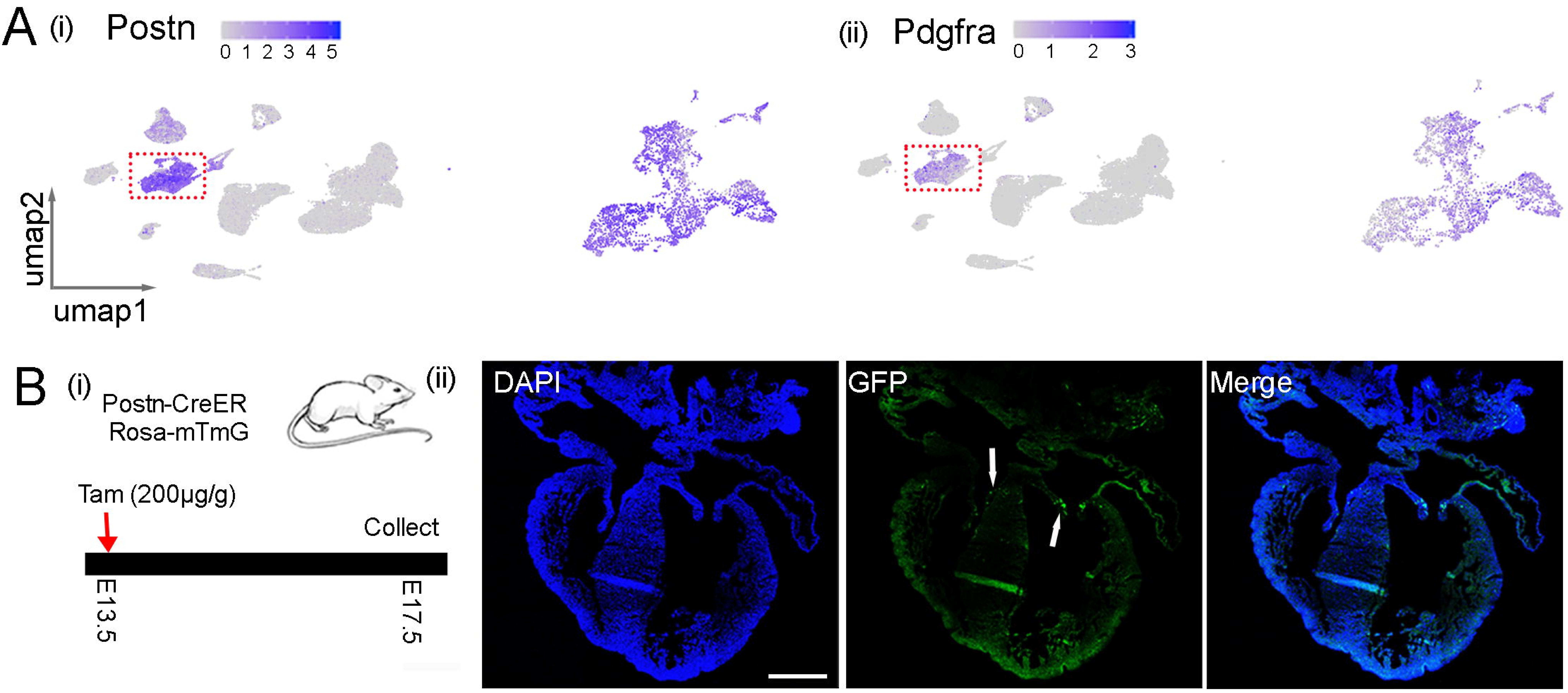

bioRxiv preprint doi: https://doi.org/10.1101/2024.09.11.612512; this version posted March 20, 2025. The copyright holder for this preprint (which was not certified by peer review) is the author/funder, who has granted bioRxiv a license to display the preprint in perpetuity. It is made available under aCC-BY 4.0 International license.

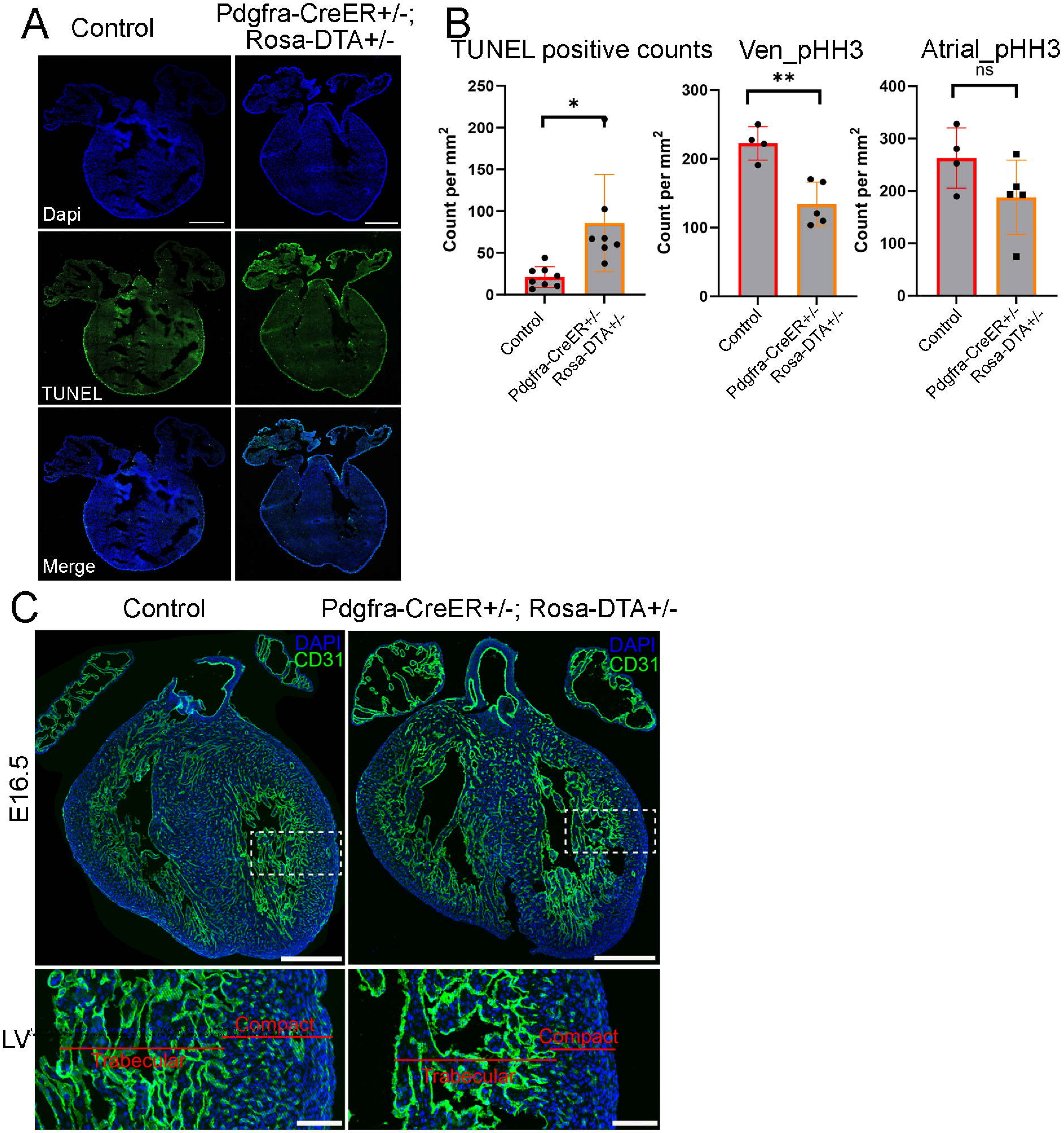
A
Control
Pdgfra-CreER+/-;
Rosa-DTA+/-
Dapi
TUNEL
Merge
B
TUNEL positive counts
Ven_pHH3
Atrial_pHH3
Count per mm2
*
**
ns
Control
Pdgfra-CreER+/-
Rosa-DTA+/-
C
Control
Pdgfra-CreER+/-; Rosa-DTA+/-
DAPI
CD31
E16.5
LV
Compact
Trabecular

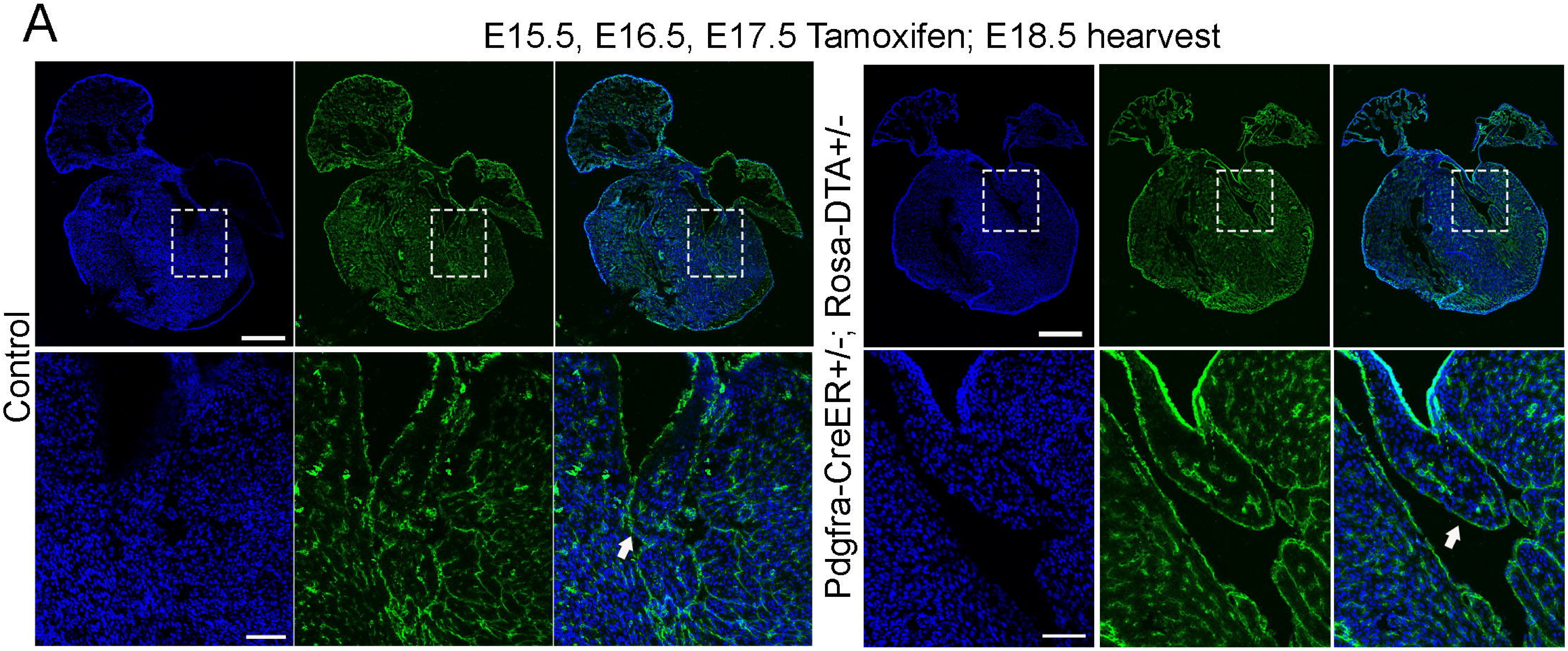

bioRxiv preprint doi: https://doi.org/10.1101/2024.09.11.612512; this version posted March 20, 2025. The copyright holder for this preprint (which was not certified by peer review) is the author/funder, who has granted bioRxiv a license to display the preprint in perpetuity. It is made available under aCC-BY 4.0 International license.

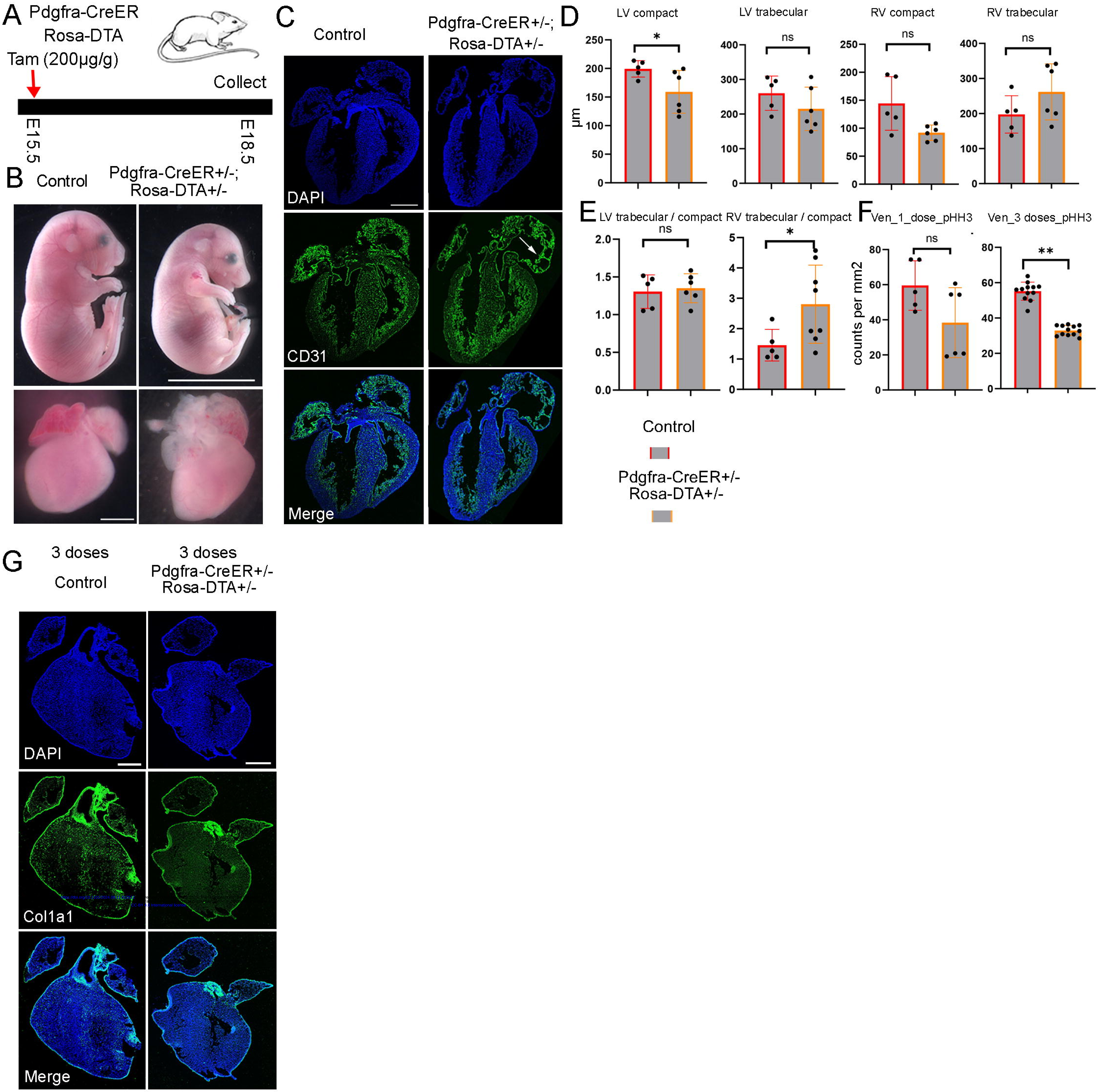
A
Pdgfra-CreER
Rosa-DTA
Tam (200µg/g)
Collect
E15.5
E18.5
B
Control
Pdgfra-CreER+/-;
Rosa-DTA+/-
C
Control
Pdgfra-CreER+/-;
Rosa-DTA+/-
DAPI
CD31
Merge
D
LV compact
LV trabecular
RV compact
RV trabecular
µm
*
ns
E
LV trabecular / compact
RV trabecular / compact
ns
*
F
Ven_1_dose_pHH3
Ven_3 doses_pHH3
counts per mm2
ns
**
Control
Pdgfra-CreER+/-
Rosa-DTA+/-
G
3 doses
Control
3 doses
Pdgfra-CreER+/-
Rosa-DTA+/-
DAPI
Col1a1
Merge

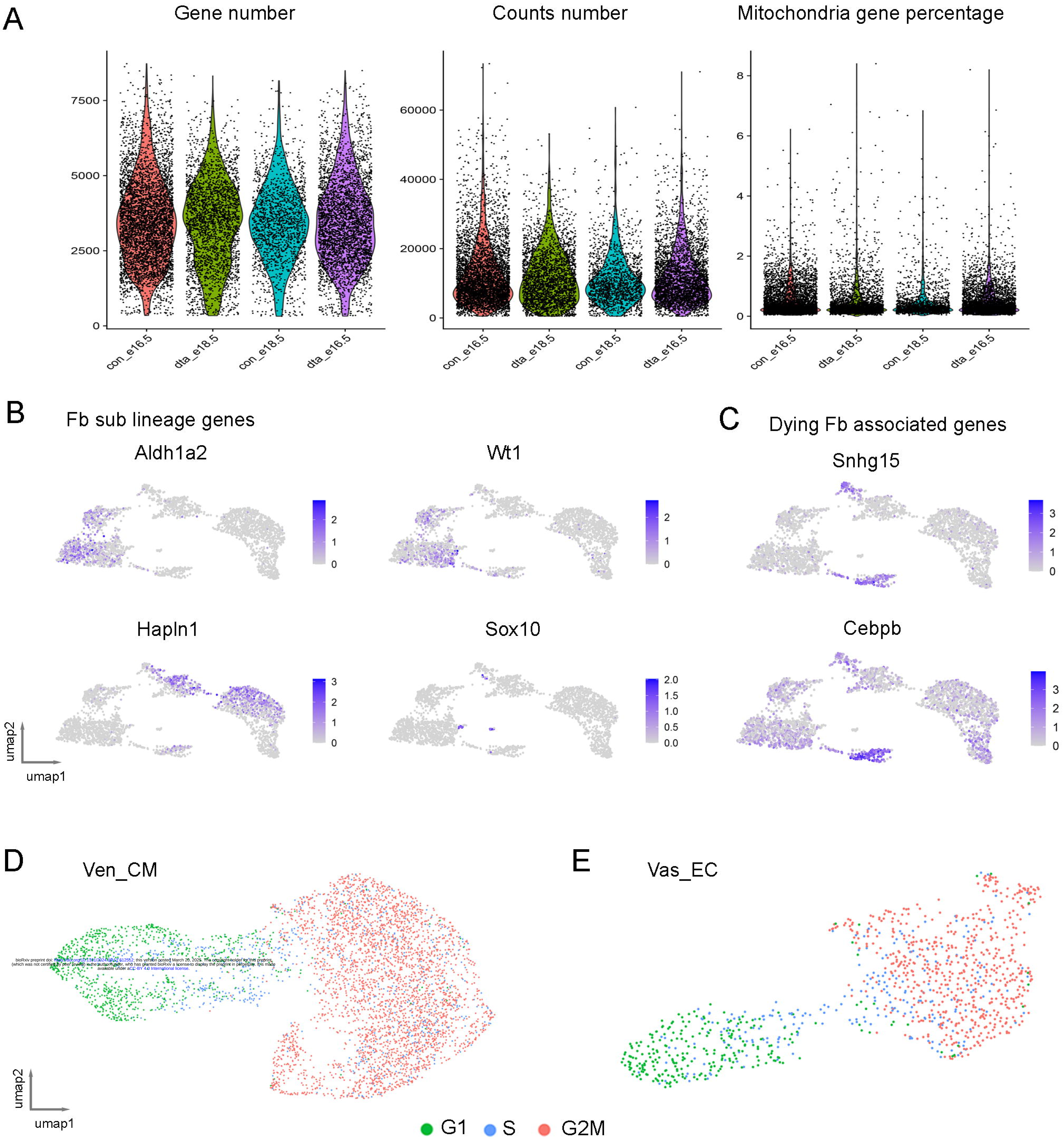

bioRxiv preprint doi: https://doi.org/10.1101/2024.09.11.612512; this version posted March 20, 2025. The copyright holder for this preprint (which was not certified by peer review) is the author/funder, who has granted bioRxiv a license to display the preprint in perpetuity. It is made available under aCC-BY 4.0 International license.

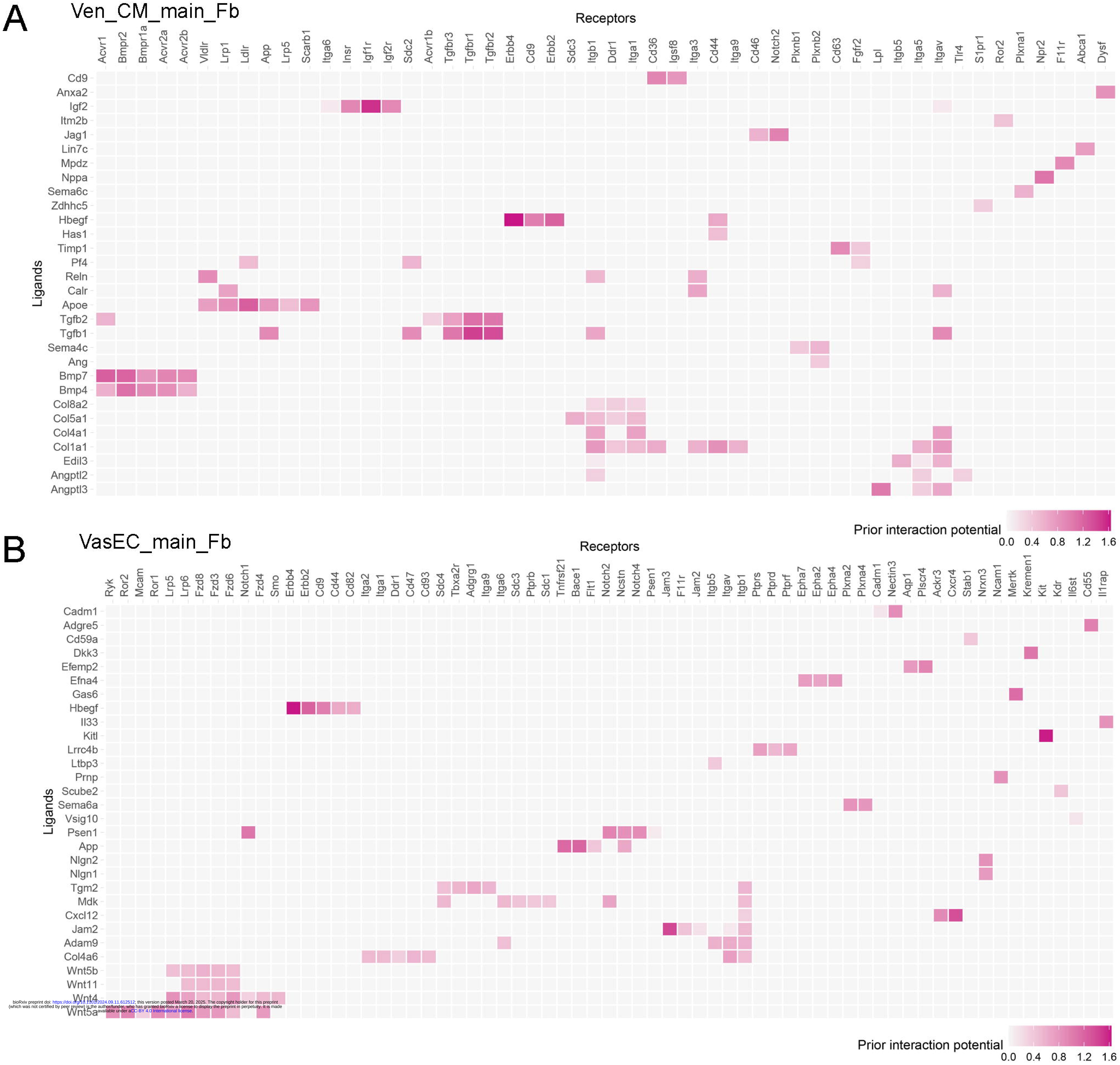

bioRxiv preprint doi: https://doi.org/10.1101/2024.09.11.612512; this version posted March 20, 2025. The copyright holder for this preprint (which was not certified by peer review) is the author/funder, who has granted bioRxiv a license to display the preprint in perpetuity. It is made available under aCC-BY 4.0 International license.

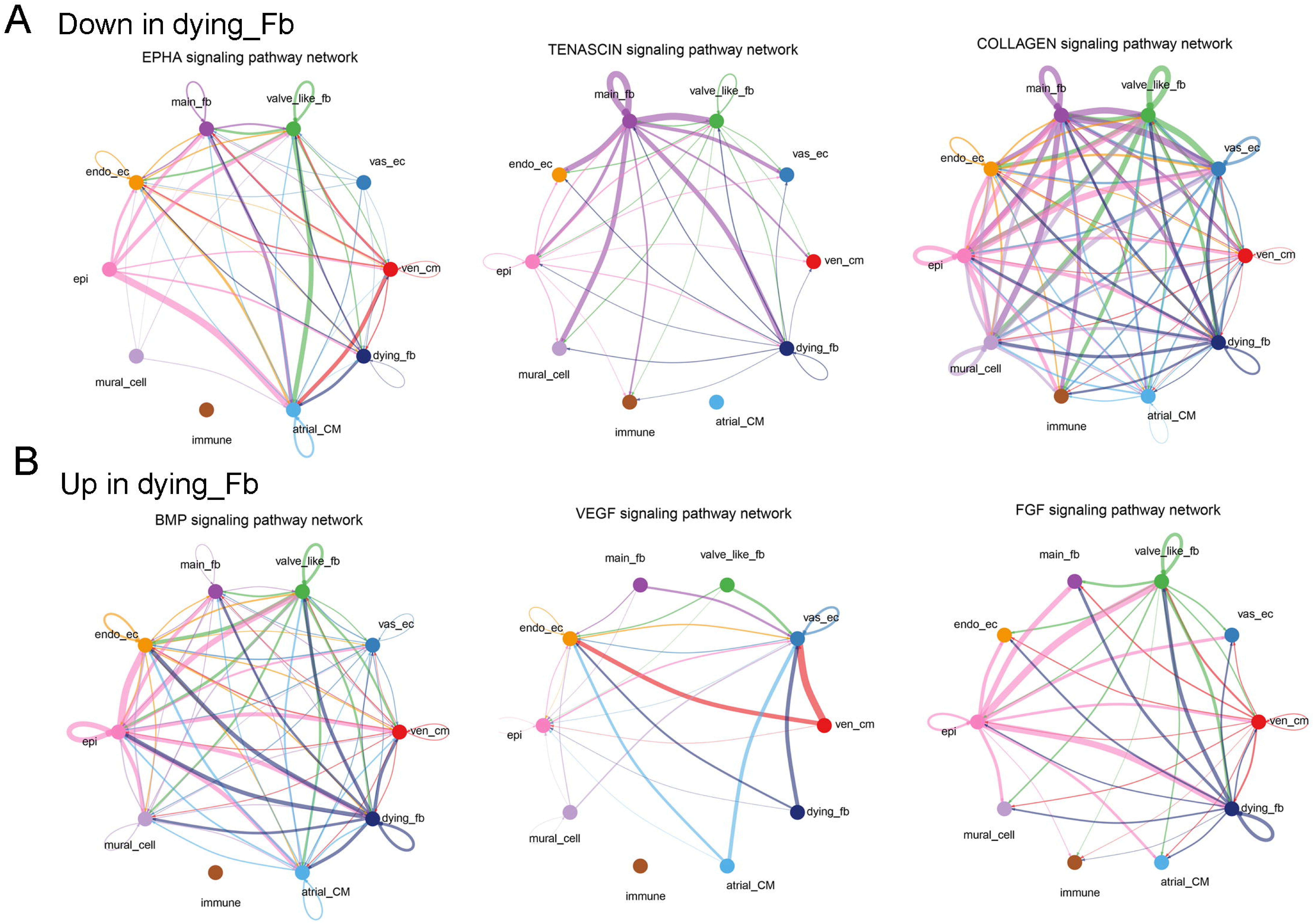

bioRxiv preprint doi: https://doi.org/10.1101/2024.09.11.612512; this version posted March 20, 2025. The copyright holder for this preprint (which was not certified by peer review) is the author/funder, who has granted bioRxiv a license to display the preprint in perpetuity. It is made available under aCC-BY 4.0 International license.

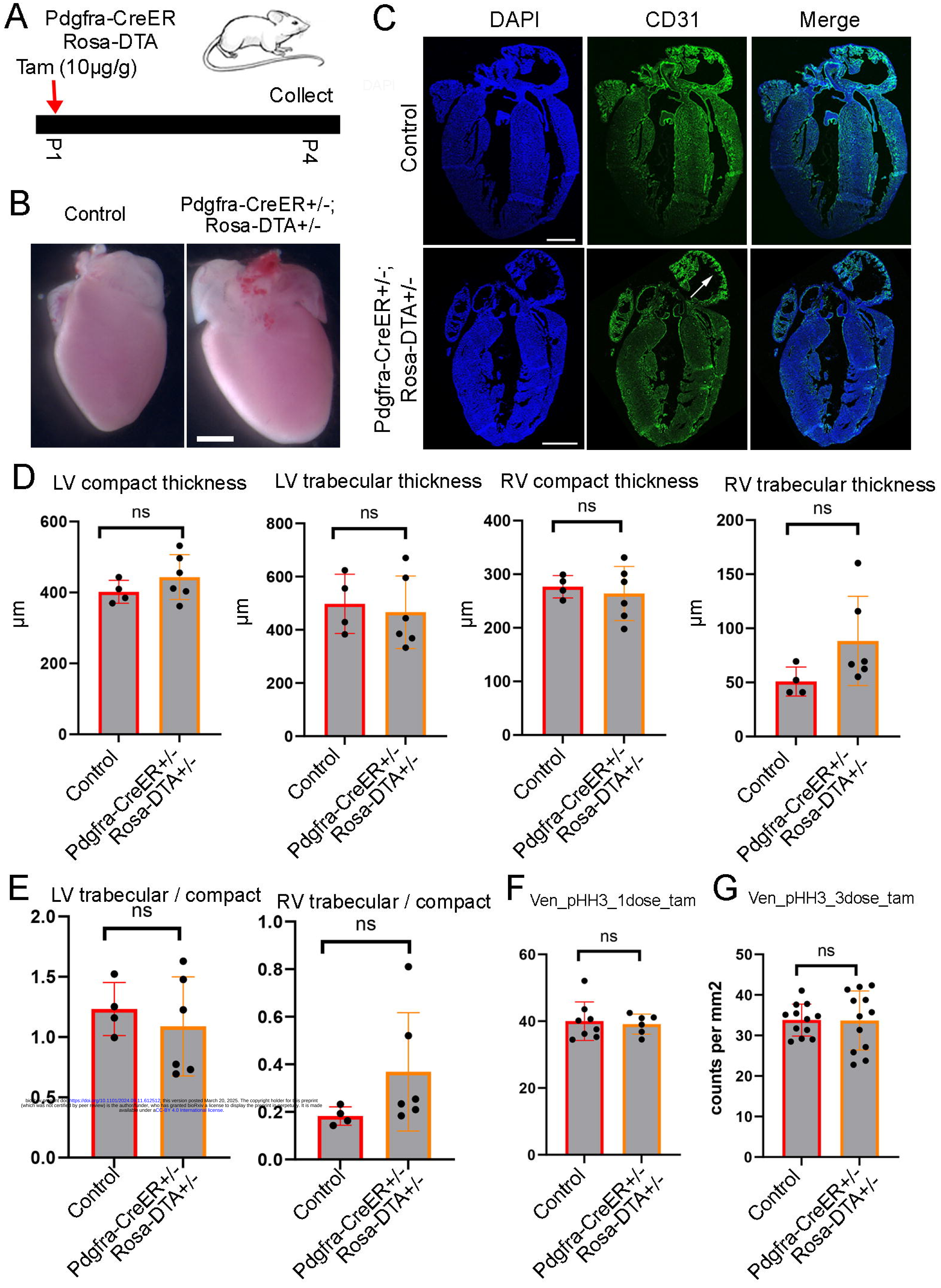

bioRxiv preprint doi: https://doi.org/10.1101/2024.09.11.612512; this version posted March 20, 2025. The copyright holder for this preprint (which was not certified by peer review) is the author/funder, who has granted bioRxiv a license to display the preprint in perpetuity. It is made available under aCC-BY 4.0 International license.

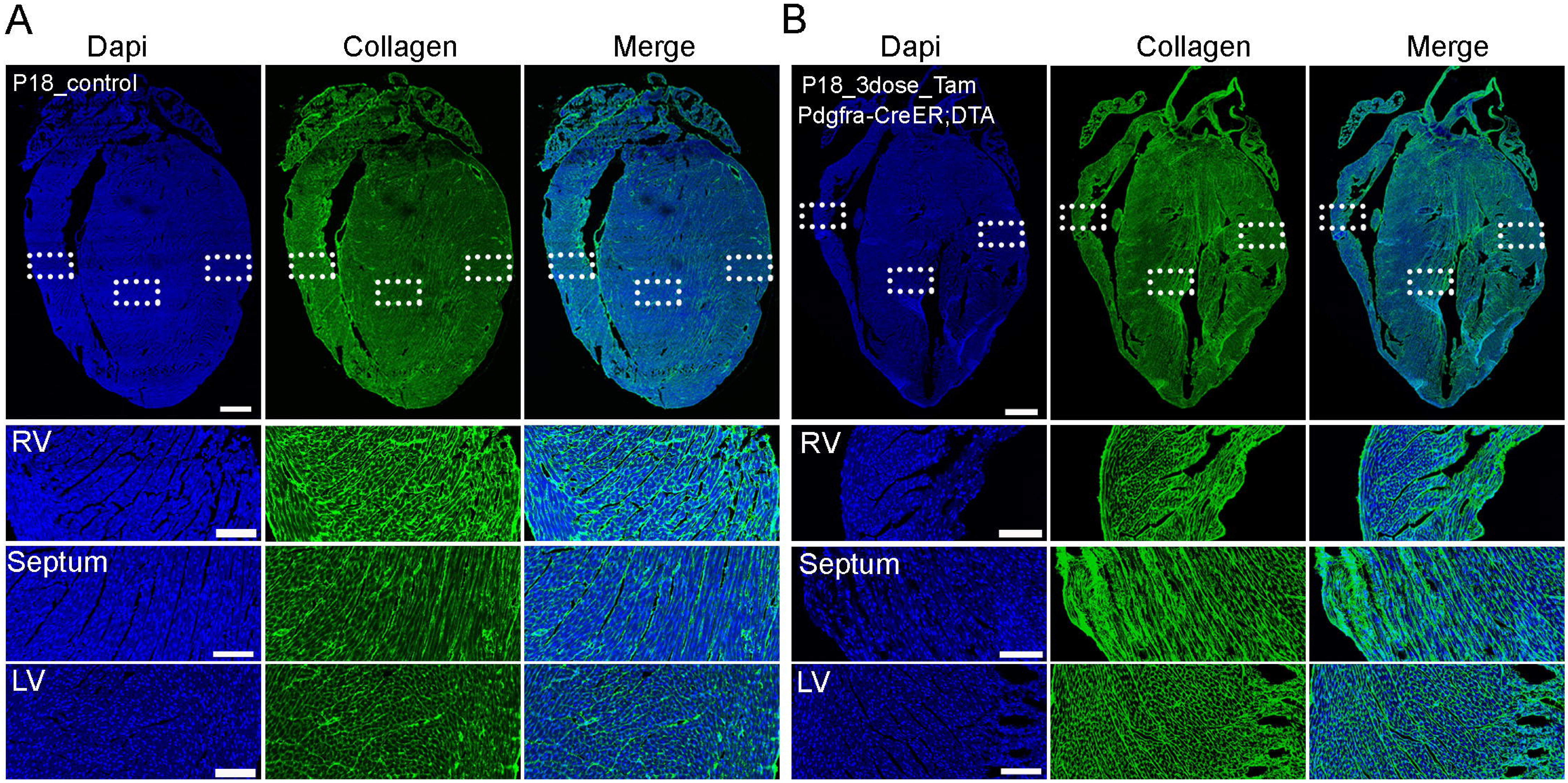

bioRxiv preprint doi: https://doi.org/10.1101/2024.09.11.612512; this version posted March 20, 2025. The copyright holder for this preprint (which was not certified by peer review) is the author/funder, who has granted bioRxiv a license to display the preprint in perpetuity. It is made available under aCC-BY 4.0 International license.

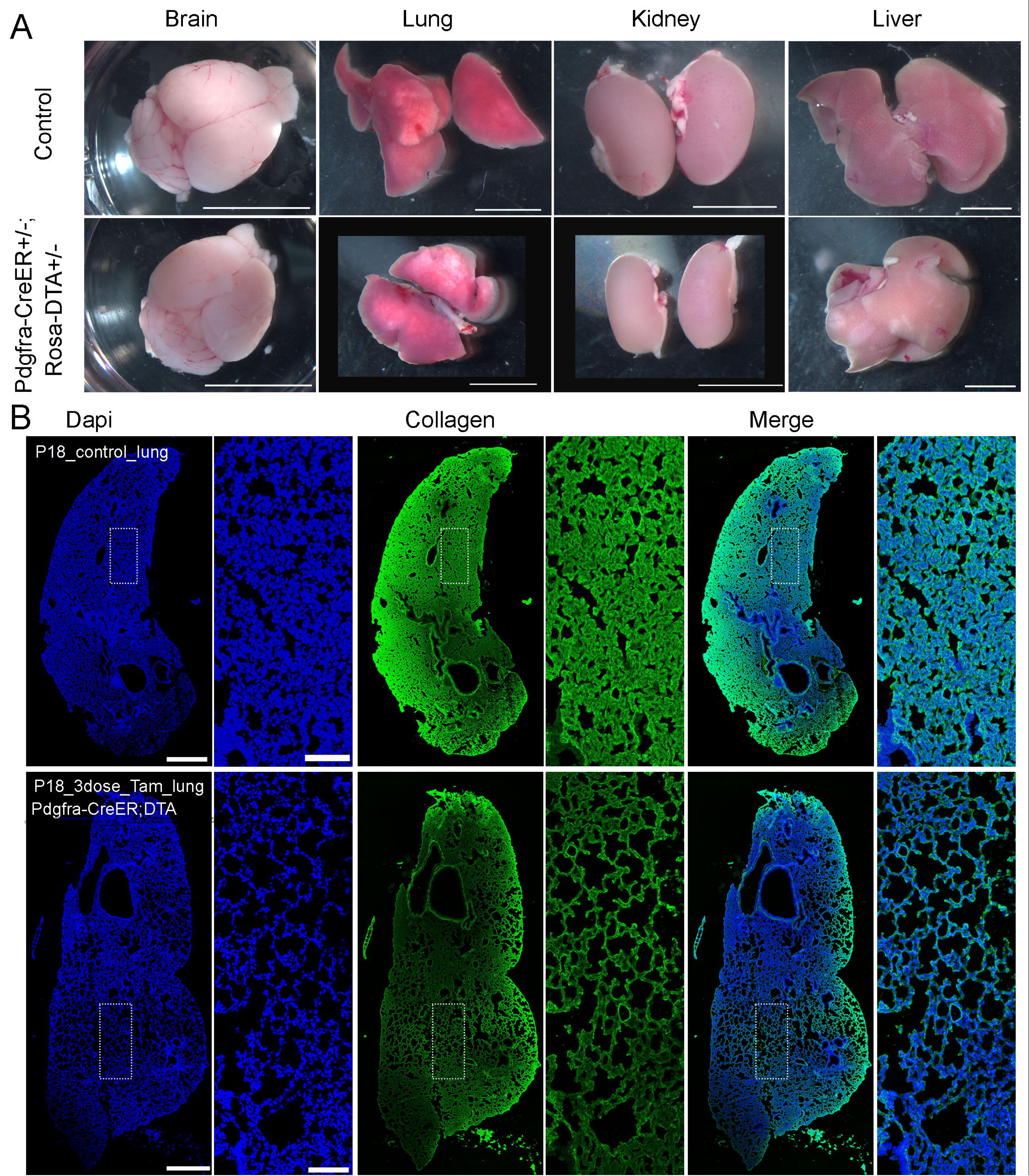

A
Brain
Lung
Kidney
Liver
Control
Pdgfra-CreER+/-;
Rosa-DTA+/-
B
Dapi
Collagen
Merge
P18_control_lung
P18_3dose_Tam_lung
Pdgfra-CreER;DTA

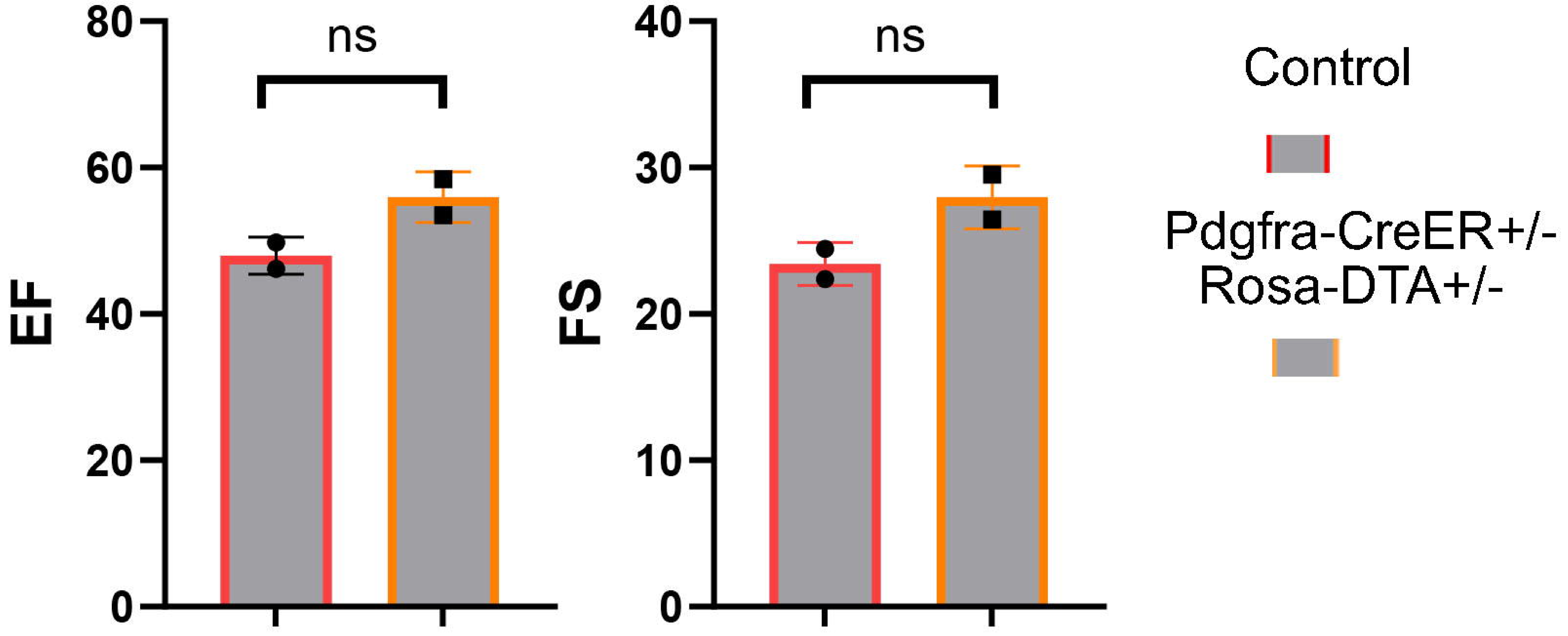

bioRxiv preprint doi: https://doi.org/10.1101/2024.09.11.612512; this version posted March 20, 2025. The copyright holder for this preprint (which was not certified by peer review) is the author/funder, who has granted bioRxiv a license to display the preprint in perpetuity. It is made available under aCC-BY 4.0 International license.
