# Supplementary material for: Cardiac Fibroblasts regulate myocardium and coronary vasculature development via the collagen signaling pathway
Source: bioRxiv. 2024 Sep 12:2024.09.11.612512. Preprint. [Version 1] doi: 10.1101/2024.09.11.612512 (PMC11418987; doi:10.1101/2024.09.11.612512)
Supplement: Supplementary file 1 [file NIHPP2024.09.11.612512v1-supplement-1.pdf]

bioRxiv preprint doi: https://doi.org/10.1101/2024.09.11.612512; this version posted September 12, 2024. The copyright holder for this preprint (which was not certified by peer review) is the author/funder, who has granted bioRxiv a license to display the preprint in perpetuity. It is made available under aCC-BY 4.0 International license.

Legends for Supplemental Figures

Supplementary Fig S1: (A) Analysis of fibroblast anatomical pattern by staining Col1a1 at different stages. Scale bar=500μm.

Supplementary Fig S2: (A, B) RNA staining of Col1a1, Actn2, and Cd31 expression at E13.5 and P3 heart sections. Scale bar=500μm and 100μm in the whole heart sections and enlarged sections, respectively.

Supplementary Fig S3: Heterogeneity analysis of cardiac fibroblasts. (A, B) The cell types and main_fb population in CD1 scRNA-seq dataset. (C, D) The cell types and main_fb population in C57BL/6 scRNA-seq dataset. (E, F) The main_fb cells labeled by stage and chamber.

Supplementary Fig S4. The expression pattern of extracellular matrix genes in the main population of cardiac fibroblasts. (A) Clustering analysis of the ECM gene expression enrichments. (B) The group of genes (G1) that were highly expressed at early staged fibroblasts in all four zones. (C) The group of genes (G2) that were highly expressed in LV and RV at late

bioRxiv preprint doi: https://doi.org/10.1101/2024.09.11.612512; this version posted September 12, 2024. The copyright holder for this preprint (which was not certified by peer review) is the author/funder, who has granted bioRxiv a license to display the preprint in perpetuity. It is made available under aCC-BY 4.0 International license.

embryonic and neonatal stages. (D) The genes (G3) that were preferentially expressed in LA and RA. (E) The genes (G4) that were highly expressed in LA, LV, and RV at neonatal stage.

Supplementary Fig S5: (A, B) The expression pattern of a group of genes (G5) that displayed expression in all four chambers of FBs (CD1 mice) at late embryonic and neonatal stages.

Supplementary Fig S6: The expression pattern analysis of extracellular matrix genes in C57BL/6 FBs. (A) Unsupervised clustering analysis of extracellular matrix genes expression in C57BL/6 FBs. (B) The groups of genes that display stage or zone-specific expression pattern.

Supplementary Fig S7: (A, B) The ligand-receptor interactions between FBs and ventricular CM or Vas_EC at each stage.

Supplementary Fig S8: (Ai, ii) UMAP plots of *Postn* and *Pdgfra* expression in cardiac cells and cardiac fibroblasts. (B) (i) The workflow to lineage trace the Postn-CreER;mTmG labeled cells. (ii) A few cells in valves and septum were labeled in Postn-CreER: Rosa26-mTmG mouse hearts. Scale bar=500μm.

Supplementary Fig S9: (A) Representative images of TUNEL staining in control and Pdgfra-CreER; Rosa-DTA hearts. (B) Quantification of TUNEL (7 sections from 2 ablated hearts and 8 sections from 2 control hearts) and pHH3 signals (5 sections from 2 ablated hearts and 4 sections from 2 control hearts). Scale bar=500μm.

Supplementary Fig S10: Quantification of the defects in control and ablated hearts with one dose of tamoxifen treatment at E15.5. (A) Diagram of the experimental procedure with one dose of tamoxifen being administered at E15.5. (B) No obvious size differences were observed in the ablated embryos and hearts compared to controls. (C) CD31 staining analysis of control and ablated hearts. (D) Quantification of the compact and trabecular myocardium thickness in LV and RV (6 sections from 2 ablated hearts and 5 sections from 2 control hearts). (E) Quantification of the ratio of trabecular to compact myocardium in LV and RV. (F) Quantification of pHH3 positive cells in control and ablated ventricles (6 sections from 2 ablated hearts and 5 sections from 2 control hearts for one dose of tamoxifen treated group, while 12 sections from 4 ablated hearts and 12 sections from 4 control hearts for three doses of tamoxifen treated group). (G) RNA staining analysis of *Col1a1* in control and ablated hearts with three doses of tamoxifen treatments. The *Col1a1* signal was dramatically reduced in the ablated hearts. * represents $p<0.05$; ** represents $p<0.01$. Scale bar=500μm.

Supplementary Fig S11: (A) QC plots of the filtered single cells including the total number of genes, total number of mRNA, and percentage of mitochondria genes. (B) The expression pattern of representative genes to identify fibroblast subpopulations. (C) The genes highly and specifically expressing in the dying_fb cells. (D, E) UMAP plots of Ven_CM and Vas_EC labeled by cell cycle phases.

Supplementary Fig S12: The predicted ligand-receptor interaction potentials between main_fb and Ven_CM or Vas_EC.

Supplementary Fig S13: The signaling pathways that were found to reduce or increase their strengths in the dying_Fb compared to the main_fb and valve_like_fb.

Supplementary Fig S14: Quantification of the defects in control and ablated hearts at neonatal stage with one dose of tamoxifen treatment. (A) Diagram of the experimental procedure with tamoxifen treatment at P1. (B) No obvious size differences were observed in the ablated hearts compared to control hearts. (C) Representative images of control and ablated hearts stained with CD31. The defects in the right atrium from ablated hearts were pointed out by an arrow.

bioRxiv preprint doi: https://doi.org/10.1101/2024.09.11.612512; this version posted September 12, 2024. The copyright holder for this preprint (which was not certified by peer review) is the author/funder, who has granted bioRxiv a license to display the preprint in perpetuity. It is made available under aCC-BY 4.0 International license.

Scale bar = 500μm. (D) Quantification of the compact and trabecular myocardium thickness in LV and RV (6 sections from 3 ablated hearts and 4 sections from 2 control hearts). (E) Quantification of the ratio of trabecular to compact myocardium in LV and RV after one dose of tamoxifen treatment (6 sections from 3 ablated hearts and 4 sections from 2 control hearts). (F) Quantification of pHH3 positive cells in ventricular in control and ablated hearts after one dose of tamoxifen treatment (6 sections from 3 ablated hearts and 8 sections from 4 control hearts). (G) Quantification of pHH3 positive cells in ventricular in control and ablated hearts after three doses of tamoxifen treatments (12 sections from 4 ablated hearts and 12 sections from 4 control hearts). * represents $p<0.05$; ** represents $p<0.01$.

Supplementary Fig S15: (A, B) Collagen accumulation in control and ablated hearts at P18. Scale bar=500μm and 150μm in the whole heart sections and enlarged sections, respectively.

Supplementary Fig S16: (A) Representative images of brain, lung, kidney, and liver from control and Pdgfra-CreER; Rosa-DTA mice at P18 with tamoxifen treatment at P3 to P5. Scale bar = 5mm. (B) Collagen staining of lung from control and ablated mice at P18. Scale bar=500μm and 150μm in the whole heart sections and enlarged sections, respectively.

Legend for Supplementary Tables:

Supplementary Table 1: Primer sequences for PLISH probes

Supplementary Table 2: The enrichment score of extracellular matrix genes in CD1 main population fibroblasts.

Supplementary Table 3: The enrichment score of extracellular matrix genes in C57BL/6 main population fibroblasts.

Supplementary Table 4: The list of ligand-receptor interactions between the main population of fibroblast and ventricular cardiomycocytes in CD1 dataset.

Supplementary Table 5: The list of ligand-receptor interactions between the main population of fibroblast and Vas_EC in CD1 dataset.

Supplementary Table 6: The list of genes that differentially expressed in control and ablated Ven_CMs at e16.5 stage.

Supplementary Table 7: The list of genes that differentially expressed in control and ablated Ven_CMs at e18.5 stage.

Supplementary Table 8: The list of genes that differentially expressed in control and ablated Vas_ECs.
